# Structure and characterisation of hydroxyethylcellulose–silica nanoparticles†

**DOI:** 10.1039/c7ra08716k

**Published:** 2018-02-08

**Authors:** Edward D. H. Mansfield, Yash Pandya, Ellina A. Mun, Sarah E. Rogers, Inbal Abutbul-Ionita, Dganit Danino, Adrian C. Williams, Vitaliy V. Khutoryanskiy

**Affiliations:** School of Pharmacy, University of Reading Whiteknights Reading Berkshire RG6 6AD UK v.khutoryanskiy@reading.ac.uk; ISIS Spallation Neutron Source, Science and Technology Facilities Council, Rutherford Appleton Laboratory, Harwell Science and Innovation Campus Didcot OX11 0QX UK; Technion – Israel Institute of Technology, Faculty of Biotechnology and Food Engineering Israel

## Abstract

Functionalising nanoparticles with polymers has gained much interest in recent years, as it aids colloidal stability and manipulation of surface properties. Here, polymer-coated thiolated silica nanoparticles were synthesised by self-condensation of 3-mercaptopropyltrimethoxysilane in the presence of hydroxyethylcellulose. These nanoparticles were characterised by dynamic light scattering, small angle neutron scattering, Nanoparticle Tracking Analysis, Raman spectroscopy, FT-IR spectroscopy, thermogravimetric analysis, Ellman's assay, transmission electron microscopy and cryo-transmission electron microscopy. It was found that increasing the amount of hydroxyethylcellulose in the reaction mixture increased the nanoparticle size and reduced the number of thiol groups on their surface. Additionally, by utilising small angle neutron scattering and dynamic light scattering, it was demonstrated that higher concentrations of polymer in the reaction mixture (0.5–2% w/v) resulted in the formation of aggregates, whereby several silica nanoparticles are bridged together with macromolecules of hydroxyethylcellulose. A correlation was identified between the aggregate size and number of particles per aggregate based on size discrepancies observed between DLS and SANS measurements. This information makes it possible to control the size of aggregates during a simple one-pot synthesis; a prospect highly desirable in the design of potential drug delivery systems.

## Abstract 2

Polymer-coated thiolated silica nanoparticles were synthesised by self-condensation of 3-mercaptopropyltrimethoxysilane in the presence of hydroxyethylcellulose.

## Graphical abstract

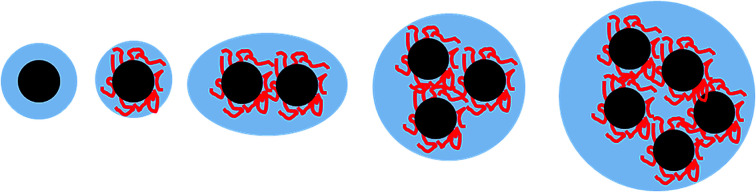

## 1 Introduction

Silica nanoparticles, ranging from 1 to 100 nm, have found numerous applications in various technical areas including drug delivery, engineering, and cosmetics.^1,2^ They can be used in adhesive formulations to enhance the thermal stability and mechanical strength of joints, or in beverage and water treatment technologies where they are employed as flocculants. Silica particles also improve the cleaning effect of some detergent formulations. Other technical applications include catalysis, coatings and polishing materials. More recently, these materials have been used in biomedical areas such as diagnostics and drug delivery.^3,4^

Thiol-functionalised silica nanoparticles have attracted particular attention due to the opportunities afforded by the SH-functional groups present on their surface. Thiolated silica is highly effective in removing mercury from aqueous solutions,^5,6^ used as a support to prepare palladium-containing catalysts,^7^ and as intermediate materials to prepare modified stationary phases for chromatography.^8^ The ease of functionalisation of these particles, for example fluorescent labelling, also provides opportunities for *in vivo* imaging and cell culture experiments.^9^

Thiolated silica nanoparticles can be easily synthesised by using 3-mercaptopropyltrimethoxysilane (MPTS) as a precursor, which undergoes either self-condensation or co-condensation with tetraethyl orthosilicate in water–ethanol mixtures, catalysed by the addition of a base.^10,11^ Recently we reported a modified synthetic approach,^12,13^ using self-condensation of MPTS in dimethyl sulphoxide which resulted in highly stable, monodisperse, sub-100 nm nanoparticles that adhered to ocular tissues through disulphide bridge formation with cysteine residues present on the mucosal surface. The presence of thiol groups on the surface of these nanoparticles also allows facile functionalisation *via* reactions with maleimide, iodoacetamide, and alkyne derivatives. Successful functionalisation of MPTS nanoparticles has been demonstrated through their fluorescent labelling, PEGylation,^14^ and POZylation.^15^

The synthesis of silica nanoparticles with prolonged stability against aggregation in aqueous media is important for their applications in a number of areas. To this end, one approach is to functionalise the surface of the particle with polymers, such as with PEG.^16–18^ However, PEG precursors used for covalent attachment to silica surfaces are often relatively expensive.

In the present study, we have successfully synthesised thiolated silica nanoparticles with a protective layer of hydroxyethylcellulose in a one-pot reaction whilst maintaining a high number of free thiol groups for further functionalisation. These particles have been fully characterised using various analytical techniques. Depending on the concentration of hydroxyethylcellulose in the reaction mixture, it was possible to prepare either individual silica nanoparticles coated with polymer chains or aggregates of nanoparticles, bridged together with polymer chains.

## 2 Experimental methods

### 2.1 Materials

3-Mercaptopropyltrimethoxysilane (MPTS), hydroxyethyl-cellulose (HEC, 90 kDa), l-cysteine hydrochloride, monopotassium phosphate (KH_2_PO_4_), sodium phosphate dibasic (Na_2_HPO_4_), deuterium oxide (D_2_O) and 5,5′-dithiobis(2-nitrobenzoic acid) (DTNB/Ellman's reagent) were obtained from Sigma-Aldrich (UK). Dimethyl sulphoxide (DMSO), and sodium hydroxide (NaOH) were from Fisher Scientific Ltd. All other reagents used were of analytical grade and purchased from Sigma-Aldrich (UK) unless otherwise stated.

### 2.2 Nanoparticle synthesis

Nanoparticles were synthesised according to the protocol of Irmukhametova *et al.* with minor modifications.^12,13^ Solutions of 0.1%, 0.5%, 1% and 2% w/v HEC were prepared in 20 mL DMSO in glass vials, and left to stir overnight at room temperature. Then, 0.75 mL of MPTS and 0.5 mL NaOH (0.5 M) were added to each HEC solution and left to stir for a further 24 hours at room temperature. The mixtures were bubbled through with air for the entire duration of the reaction to facilitate the formation of disulphide bridges.^12,13^ Samples were then purified by dialysis, using cellulose membranes with a molecular weight cut-off of 12–14 kDa (Medicell Int. Ltd., UK). Samples were dialysed against 5 L deionised water (changed every 2 hours) for 48 hours. Following synthesis, 1 mL of each nanoparticle suspension was frozen, and placed in a Heto PowerDry LL3000 freeze-dryer to obtain a solid sample used for further analyses. The yields of nanoparticles were determined gravimetrically after particles purification using dialysis and subsequent lyophilisation.

### 2.3 Ellman's assay

Ellman's assay was used to determine the concentration of thiol groups on the surface of nanoparticles.^12,13,19^ Freeze-dried nanoparticles were rehydrated in a solution of phosphate buffer (pH 8, 0.5 M) at 0.3 mg mL^−1^. The particles were left to incubate for 1 hour prior to the assay. Following this, 500 μL aliquots of particle dispersions were placed in Eppendorf tubes (5 for each concentration of HEC), to which 500 μL of DTNB solution (0.3 mg mL^−1^) was added. Samples were left to incubate for 2 hours. Alongside this, standards of cysteine–HCl were used in the concentration range 25 to 225 μmol L^−1^. Again, samples were prepared in phosphate buffer, and treated exactly the same as the test samples. Following the incubation, 200 μL from each Eppendorf tube was placed into a single well of a 96 well-plate. 3 samples were taken from each Eppendorf. Absorbance at 420 nm was then measured using a BioTek Epoch plate reader, and the concentration of free thiol calculated based on the absorbance generated from the standards. Data are presented as mean ± standard deviation of the 5 aliquots taken for each HEC concentration.

### 2.4 Dynamic light scattering

Dynamic light scattering (DLS) was used to measure the hydrodynamic diameter and polydispersity of the synthesized nanoparticles. Samples were diluted by a factor of 1 : 10 into deionised water, to form a clear suspension, placed into low-volume cuvettes (Fisher Scientific), and measured using a Zetasizer nano-ZS instrument at 25 °C, with a refractive index of 1.471. Individual recordings were measured three times, for three repeat readings. Data are presented as mean ± standard deviation for the three repeats.

### 2.5 Nanoparticle tracking analysis

Nanoparticle tracking analysis (NTA) employed a NanoSight LM10, with a LM14 top-plate attached, syringe pump, and a 533 nm laser (Malvern Instruments, UK). Prior to the experiments, 10 000 fold dilutions were made for each nanoparticle suspension into ultrapure water (18 MΩ cm^−1^). Samples were then injected into the top-plate using a NTA syringe pump, and kept at a constant flow of 20 AU for all recordings. 3 × 60 second videos were recorded for each sample and repeated 3 times for each sample. All recordings were made at 25 °C, and the viscosity was that of water (0.89 cP). The mean and standard deviation were then calculated from the triplicates.

### 2.6 Raman spectroscopy

Freeze dried nanoparticles were placed in glass vials and FT-Raman spectra were recorded for each sample using a FT-Raman NXR 9600 Raman spectrophotometer (Thermo Scientific). 2000 scans were recorded and averaged for each sample at a resolution of 4 cm^−1^.

### 2.7 Thermogravimetric analysis

Thermogravimetric analysis (TGA) was performed with a TGA Q50 (TA instruments, UK). Dry samples were placed into clean ceramic pans, and thermal decay measured from room temperature (25 °C to 1000 °C), at a temperature ramp of 10 °C per minute in a dynamic nitrogen environment (20 mL min^−1^ flow). Data was analysed using TA universal analysis software, and presented at% weight change as a function of temperature.

### 2.8 FT-IR spectroscopy

Fourier transformed infra-red (FR-IR) transmittance spectra were recorded for freeze dried samples of nanoparticles using a FT-IR Spectrum 100 (Perkin Elmer) spectrophotometer. Spectra are the average of 32 scans, over a range of 500–4000 cm^−1^ at a resolution of 4 cm^−1^.

### 2.9 Transmission and cryo-transmission electron microscopy

TEM images of the HEC coated nanoparticles were obtained with the use of a Philips CM20 Analytical TEM at 80 kV accelerating voltage. Samples were prepared by placing a drop of nanoparticles in aqueous suspension onto carbon coated grids for 1 minute and then exposed to a 1% uranyl acetate solution before being dried and placed in the instrument.

Vitrified specimens were prepared at a controlled temperature and at water saturation in the Vitrobot (FEI, Netherlands), and kept in liquid nitrogen until examination. Cryo-TEM analysis was done with a Tecnai T12 G2 TEM (FEI, Netherlands) operating at 120 kV. Images were recorded digitally on a Gatan UltraScan 1000 camera using the DigitalMicrograph software (Gatan, U.K.). Images are recorded in the low-dose imaging mode to minimize beam exposure and electron-beam radiation damage.^20,21^

### 2.10 Small angle neutron scattering (SANS)

A 2 mL dispersion of each type of HEC-functionalised nanoparticle and parent thiolated silica nanoparticles was dialysed against D_2_O using a dialysis membrane, with a molecular weight cut off of 2 kDa (Medicell International, UK). D_2_O was replaced every 3 hours with a total of three times to ensure close to complete dispersion in D_2_O.

SANS experiments used the Sans2d neutron diffractometer at ISIS, UK. The instrument set up and incident wavelength employed a *Q* range of 0.004–0.4 Å^−1^. *Q* is defined in eqn (1);1
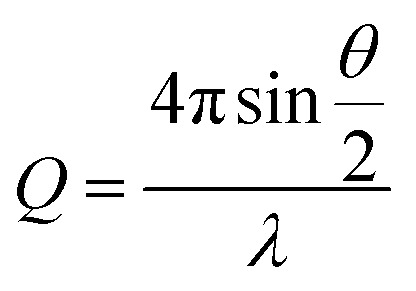
where *θ* is the scattering angle of the neutron beam, and *λ* incidence wavelength. Data were collected on a ^3^He detector, set at 4 m from the sample, and offset by 150 mm vertically and 180 mm laterally. All samples were placed into 2 mm path-length quartz cuvettes without any further dilution from stock. Each raw scattering data set was corrected for the detector efficiencies, sample transmission and background scattering before being reduced using instrument specific software, Mantid,^22^ and placed on an absolute scale using a reference material (a solid blend of hydrogenous and perdeuterated polystyrene). All samples were measured for the time required to obtain data of high statistical precision. Data were then modelled using the SASview programme (see ESI† for details).

Initially the parent thiolated silica sample (Y1) was modelled to a sphere only, as it was synthesised without the presence of any polymer, and therefore provided baseline information including the scattering length density (SLD) and radius of the particle core, which was used for further analyses. Following this, the parent particle was also fitted to a core–shell model, along with the remaining data for samples decorated with HEC (Y2–Y5). Data obtained from particle characterisation (including radius, polydispersity and density) were used for the fitting.

## 3 Results and discussion

### 3.1 Particle size characteristics

Thiolated silica nanoparticles were synthesised in the presence of hydroxyethylcellulose (HEC), a known thickening agent. This led to the formation of nanoparticles varying in size and with altered surface functionality dependent on the concentration of HEC used, but retained a high concentration of free thiol groups available for further functionalisation, *e.g.* for fluorescent labelling. Immediately following synthesis, particles were sized using three techniques; dynamic light scattering (DLS), nanoparticle tracking analysis (NTA), and TEM.

**Fig. 1:**
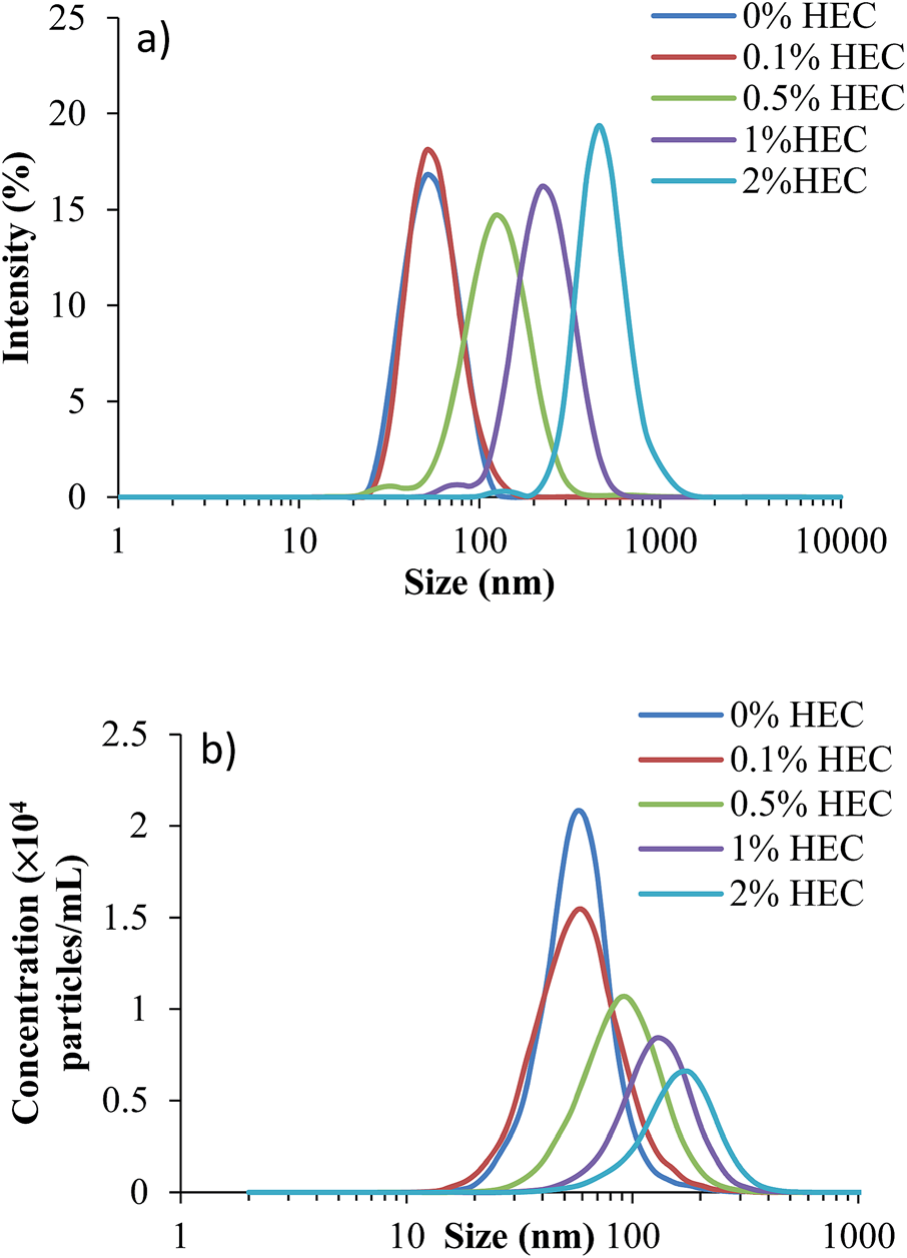
Size distributions for silica nanoparticles functionalised with 0% w/v HEC (dark blue), 0.1% w/v HEC (red), 0.5% w/v HEC (green), 1% w/v HEC (purple), and 2% w/v HEC (blue). (a) Shows data generated from dynamic light scattering, whilst (b) shows data generated from NTA.

DLS is a well-established and widely used technique for determining the size and polydispersity of nanoparticles within a sample. The size distribution profiles for the silica nanoparticles (parent and HEC-functionalised) are in Fig. 1a with numerical values in Table 1. As can be seen, increasing the concentration of HEC added to the reaction mixture increases the particles hydrodynamic radius, suggesting the hydroxyethylcellulose is responsible for this effect.

**Table 1:** Summary of the characteristics of silica nanoparticles functionalised with varying concentrations of HEC. Data show the means ± standard deviation

Sample	% mass of HEC (w/v)	Yield (%)	Hydrodynamic radius, nm (DLS)	PDI (DLS)	Hydrodynamic radius, nm (NTA)	Particle radius, nm (TEM)^a^	Grafting^b^ density (μg nm^−2^)	Surface thiol content (μmol g^−1^)
Y1	0	58 ± 2	25 ± 1	0.102	37 ± 1	15	—	552 ± 185
Y2	0.1	61 ± 3	27 ± 2	0.143	50 ± 5	19	0.058	348 ± 41
Y3	0.5	60 ± 1	54 ± 2	0.210	59 ± 3	19	0.064	267 ± 83
Y4	1	62 ± 3	97 ± 11	0.236	78 ± 3	17	0.202	189 ± 24
Y5	2	65 ± 4	190 ± 30	0.241	99 ± 4	20	0.144	207 ± 20

a Exemplary TEM images are shown in Fig. SI1.

b It should be noted that grafting density calculations used the surface area of an individual nanoparticle obtained by TEM analysis, as this was most accurate to the raw particle size. It should also be noted, that given the likely presence of free HEC in the dispersion, there may be some error in these calculations.

A complimentary particle sizing technique, NTA, was also used. NTA maps the movement of individual particles over a given time and generates a diffusion coefficient based on the number of pixels the particles cross.^23^ From this diffusion coefficient, a particle size is obtained using the Stokes–Einstein equation (eqn (2));2
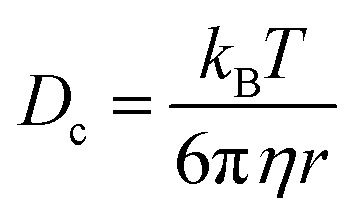
where *k*_B_ is the Boltzmann constant, *T* is the temperature (K), *η* is the viscosity (kg s) of the suspension solution, and *r* is the particle radius. By combining DLS and NTA data, it is possible to provide a greater accuracy of particle size. It is worth noting that although both DLS and NTA determine the particles *r*_H_, the method used to determine the values is different.

DLS is a light scattering technique where a laser is shone through a suspension of particles which causes the laser light to scatter (Rayleigh scattering). Fluctuations in the scattering, due to particles moving under Brownian motion, are measured at specific time intervals, which are then fitted to an autocorrelation function. A diffusion coefficient is then determined using Mie theory (based on this autocorrelation function and the refractive index of the sample), followed by a particle size using the Stokes–Einstein equation.

NTA on the other hand is a particle tracking technique, and allows for a diffusion coefficient to be measured by analysing the movement of individual particles in a specific environment. By tracking individual particles, undergoing random Brownian motion from frame to frame, the average spatial displacement of the particles per unit time can be calculated, and this displacement can be related to the hydrodynamic diameter of the particles through the Stokes–Einstein equation (eqn (2)). Although translational Brownian motion is a three-dimensional process, it is possible to use a one, two, or three dimensional diffusion coefficient to determine a particles hydrodynamic diameter as described by eqn (3):3
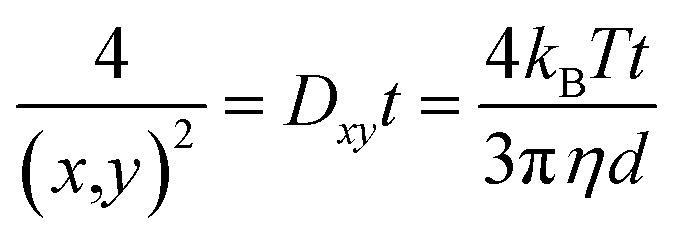


It should be noted that there is no assumption of 2-dimensional movement of particles. All particles are assumed to be moving freely in all 3 dimensions while the measurement is sampling the projection of each *x*, *y* and *z* component of that movement onto the *xy* observation plane.

As can be seen from the size distributions, there are discrepancies between the results generated by each sizing technique. These are quantified by changes in the average sizes observed (Table 1). The decrease in concentration observed by NTA is likely due to the increasing size of particles, which form aggregates. The larger aggregates are formed in solutions with greater concentrations of HEC, where the macromolecules may act as bridges to bind individual thiolated silica nanoparticles. The larger size of these aggregates leads to a smaller number in suspension, which reduces their total concentration measured by NTA.

Table 1 shows additional DLS and NTA data, along with the particle yield produced and concentration of reactive thiol groups on their surface (as determined by Ellman's assay). As can be seen, yields from the synthesis were relatively similar, and no significant difference was noted (*p* > 0.05). This could be related to the presence of free HEC, which could not be removed by dialysis. In terms of sizing (based on the *z*-average produced by DLS and the mean particle size from NTA), there is a discrepancy. This is due to the way the data is collected in each case. The *z*-average from DLS is a mean particle size based on the intensity, and is bias towards a larger particle size (due to increased scattering from larger particles compared to smaller particles); however, the mean particle size recorded by NTA is the mean of all the particles measured, and therefore shows no bias towards polydisperse samples. Fig. 1 shows the size distributions for both DLS and NTA (a and b respectively), showing a range of the particle sizes in the suspension. The peak of each curve respectively corresponds to the hydrodynamic size quoted in the table.

It is also apparent from Table 1 that polydispersity index (PDI) increases as more HEC is added to the reaction mixture (from 0.102 with no HEC to 0.241 with 2% HEC). This suggests either particle aggregation or free HEC was present in the particle dispersion.

### 3.2 Particle surface characteristics

**Fig. 2:**
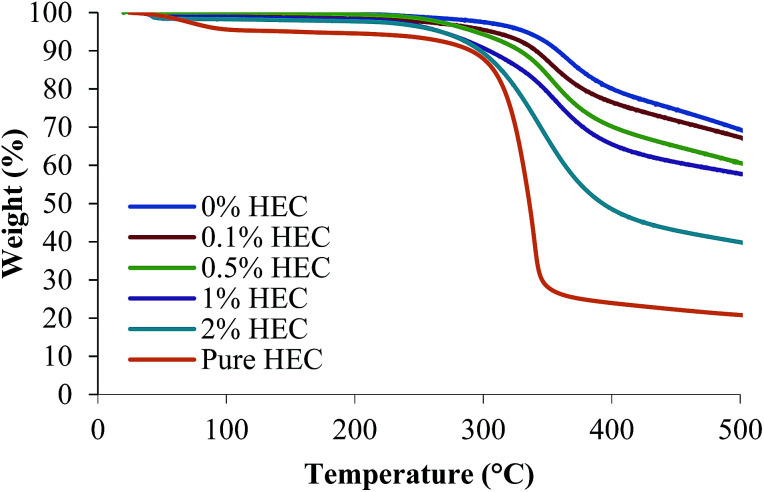
Thermal decomposition of thiolated and HEC-functionalised nanoparticles, and pure HEC measured using TGA.

The surface functionality of the particles was assessed using FTIR, Raman spectroscopy and TGA. Fig. 2 shows the thermal decomposition of the raw unfunctionalised nanoparticles, the polymer alone, and the functionalised particles. As expected, increasing the amount of HEC in the reaction mixture increases the weight loss from the sample, demonstrating that more HEC is present. The silica synthesised in the presence of 0.1% w/v HEC (red) has a similar thermal decomposition to the unfunctionalised silica (blue), as would be expected. However the % weight loss for this sample appears lower at the extreme temperatures than the unfunctionalised silica. From Table 1, it is clear that both Y1 and Y2 are of similar sizes. Therefore, it could be possible that only a few macromolecules of HEC are bound to silica surface in the case of Y2.

By calculating the weight loss in each sample and subtracting it from the mass difference in the unfunctionalised silica nanoparticles, it is possible to determine the grafting density of HEC in the particle dispersion (Table 1). It can be seen that increasing the amount of HEC in the reaction mixture increases the grafting density on the particle surface. The exception to this is Y5, where (when compared to Y4) a decrease in grafting density can be observed, from 0.202 to 0.144 μg nm^−2^. Although the % mass loss increases in the 2% HEC, the grafting density appears lower. A likely reason for this is that the HEC is more densely incorporated into the internal structure of the particles, and thus more protected from thermal degradation compared to HEC bound loosely on the particle surface.

**Fig. 3:**
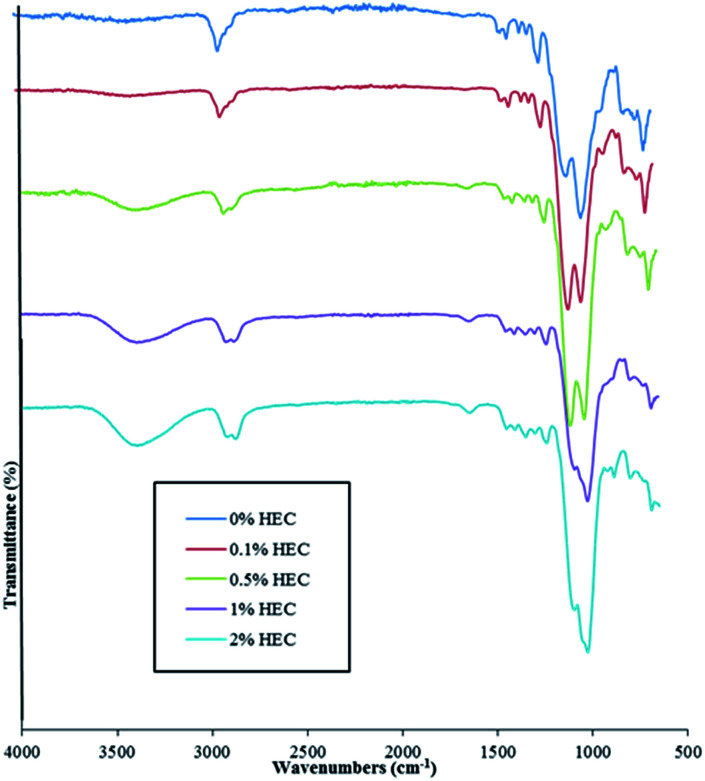
FT-IR spectra of nanoparticles prepared from reaction mixtures with different HEC content.

**Fig. 4:**
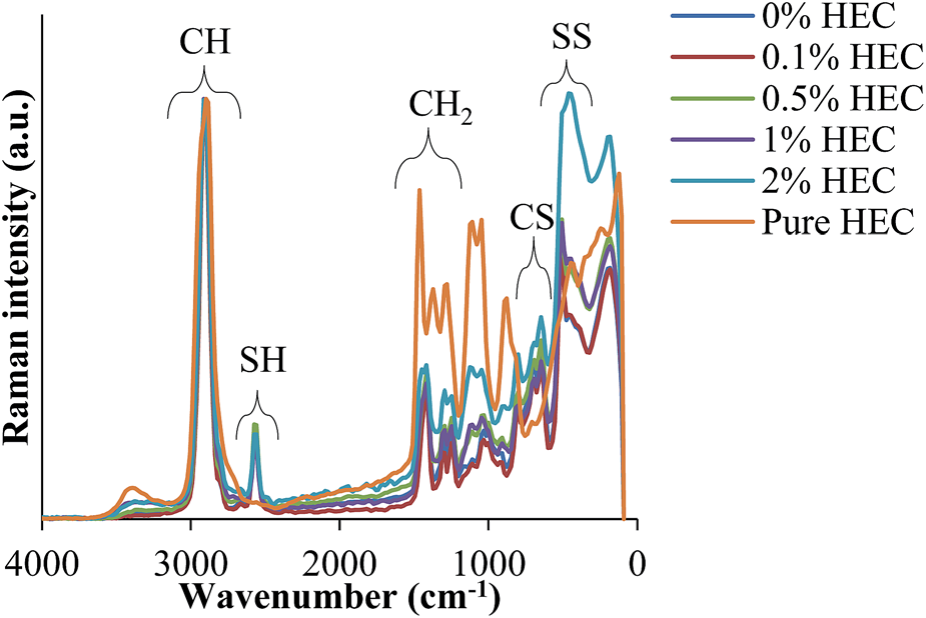
FT-Raman spectra of nanoparticles prepared from reaction mixtures with different HEC content. Spectrum of pure HEC is also included for comparison.

FT-IR and FT-Raman spectroscopies confirm the presence of HEC in the particle suspension. Spectra are shown for both FT-IR and Raman in Fig. 3 and 4, respectively.

As shown in Fig. 3, adding HEC into the reaction mixture introduces a broad band in the region of 3200 cm^−1^ to 3600 cm^−1^. This is attributable to the O–H groups present in HEC. As the concentration of HEC in the nanoparticles increases, a second band at 2880 cm^−1^ and 2900 cm^−1^ becomes more distinctive and provides further evidence that increasing the amount of HEC in the reaction mixture increases polymer loading onto the nanoparticles. Another feature evident in the spectra for nanoparticles that contain HEC (as well as pure HEC) is the band at around 1640 cm^−1^, attributable to bound water within the cellulose.^24^ The large peak observed at 1000–1200 cm^−1^ arises from the Si–O–Si linkage, as seen in other publications using the same particles.^14,15^

Fig. 4 shows FT-Raman spectra of HEC-functionalised silica nanoparticles. These data demonstrate the presence of both thiol groups (at 2559 cm^−1^) and disulphide bonds (at 490 cm^−1^) in the nanoparticles synthesised from MPTS. It also exhibits many more similarities to the spectra recorded for uncoated organosilica as reported in previous work.^14,15^ The presence of disulphide bonds results from thiol groups being oxidised during the synthesis procedure.^12,13^

The Raman spectra were normalised to the maximum intensity of the C–H band. This allows the data from each of the batches to be overlaid to facilitate comparisons between nanoparticles synthesised with varying concentrations of HEC. Fig. 4 illustrates that the spectra differed significantly between the shifts of 1500 cm^−1^ and 0 cm^−1^. These differences appear to follow a trend that may be caused by the increasing amount of HEC. The differences are especially pronounced between 1500 cm^−1^ and 1000 cm^−1^. Indeed, it was found that as the intensity of –SH groups decreases, the intensity of aliphatic C–C stretches increases. Given that C–C exists mostly in the HEC, and SH in the silica, this confirms this hypothesis.

From the spectroscopic and thermal data, it is clear that the particle size increase resulting from increasing amounts of HEC in the reaction is due to the presence of this polysaccharide on the particle surface, or bound into the particle core. As the volume of the nanoparticle precursor is the same, it appears that the HEC macromolecules are binding into the core of the particle, causing a larger cluster for which the particles can form around. By doing so, the concentration of surface thiol groups is lower (shown by a smaller peak at 2550 cm^−1^ in the FT-Raman spectra, and molar concentration shown by Ellman's assay).

### 3.3 Particle structure

**Fig. 5:**
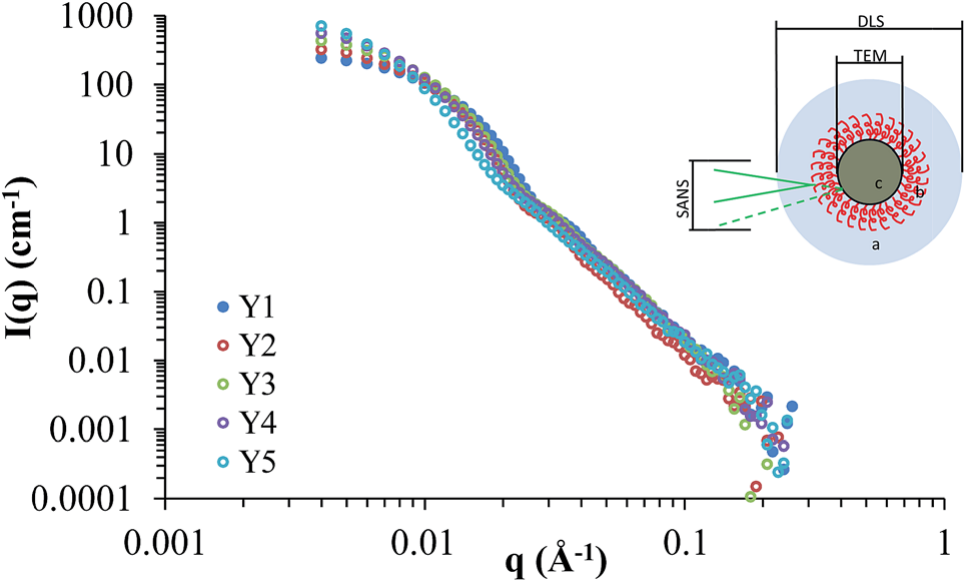
Scattering cross-sections for Y1, Y2, Y3, Y4, and Y5. The insert demonstrates differences in sizes observed by DLS, TEM, and SANS.

Small angle neutron scattering (SANS) provided information on the internal and external structure of the nanoparticles, along with their sizes and potential interactions. Previously we have characterised silica nanoparticles functionalised with poly(ethylene glycol) and poly(2-oxazoline)s using SANS, in order to confirm their structure.^14,25^ Here we used SANS to assess how the addition of HEC influences the particle size and core–shell structure. The scattering cross-sections for Y1–Y5 are shown in Fig. 5, and the fits (along with details on the form factors used) can be viewed in the ESI (Fig. SI2†). It should be noted that the SANS experiments used a different batch of nanoparticles than those summarised in Table 1, however, no significant difference was found between the size and coverage data between the two populations (*P* > 0.05). Given the uncertainty of how the silica and HEC are interacting (*e.g.* HEC functionalised onto the surface, or through the core), the SLD for the core and shell were left as floating variables in the fitting process.

Table 2 provides a summary of the sizes for each population of particles used in this study, based on modelling the SANS data to a core–shell sphere (see ESI† for a full breakdown). Data obtained from previous characterisation techniques (TEM for the core particle size, and DLS for the hydrodynamic size, Table 1), was used as a guideline in the fitting process. The core-radius and shell thickness parameters were obtained directly from modelling the SANS data. The total diameter was calculated by adding together the radius and shell thickness (to obtain total radius), and multiplied by 2. The values calculated matched the values obtained by TEM (Table 1), confirming their accuracy. The value for number of particles per aggregate was estimated from the aggregate volume (by DLS) divided by volume of hydrated particles at each HEC concentration (from SANS data plus an estimated 20 nm for the hydration layer around each particle). Further details (and caveats) of the estimation can be found in the ESI.† For the purposes of this discussion, “aggregate size” refers to the hydrodynamic diameter obtained by DLS, and “particle size” refers to the size calculated from SANS modelling.

**Table 2:** Core radius and shell thickness of HEC-functionalised silica nanoparticles as determined by fitting SANS data to a core–shell model, along with the calculated number of particles per aggregate as described in ESI

Sample	Core radius (nm)	Shell thickness (nm)	Total diameter (nm)	Estimated particles per aggregate	
Y1	15.2	0.5	31	1	
Y2	17.2	0.5	35	1	
Y3	17.9	3.0	41	6	
Y4	14.6	3.0	35	44	
Y5	17.5	3.4	42	230	

Comparing the particle sizes obtained for the unfunctionalised silica (*i.e.* particles with no HEC present, Y1), generated by TEM/SANS to that of DLS, a discrepancy of 19 nm can be observed (31 nm in SANS/TEM, and 50 nm in DLS). Previously we have reported this to be due to the hydration shell surrounding the particle; DLS provides a radius of hydration, whereas SANS and TEM will provide the raw particle size (*i.e.* size without the hydration shell). A hydration shell thickness of 10 nm, similar to the value obtained here, was observed.^14,25^

From the DLS data and TEM images for the remaining samples (Y2–Y5), it is clear that the particles synthesised with increasing amounts of HEC have a tendency to form larger aggregates. It is possible that, given the increasing amount of HEC in the solution, that several particles are interacting with a single HEC chain, which acts as a bridge, thus leading to increased aggregation. By correlating the DLS and SANS data, it is possible to determine the number of particles per aggregate (taking into account a 10 nm radius hydration shell around each particle (Table 2, and ESI†)). Interestingly, a relationship was found between aggregate size (and the consequent number of particles per aggregate) and the mass of HEC used in the reaction (Fig. SI3†).

**Fig. 6:**
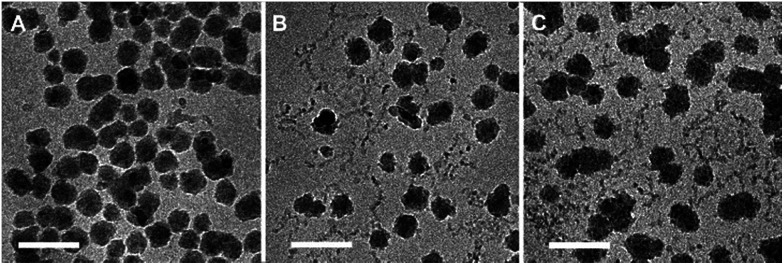
Cryo-TEM images of thiolated silica nanoparticles functionalised with (a) 0.1% w/v HEC, (b) 0.5% w/v HEC, and (c) 1% w/v HEC. All scale bars = 100 nm.

**Fig. 7:**
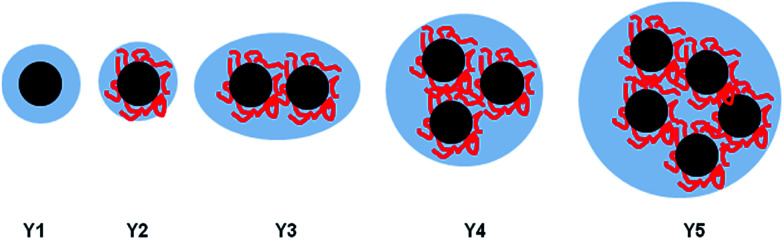
Illustration of the increased packing of particles in aggregates as HEC content increases in the reaction mixture.

The determined aggregate volume appears to correlate well with the cryo-TEM images shown in Fig. 6. Given that both the particles per aggregate and aggregate size correlate very well with the concentration of HEC (with *R*^2^ values of >0.99), it is likely that HEC is interacting with growing particles, and anchoring several particles together. Given that the values appear relatively consistent, with little error within the same system (from the small PDI values from DLS), it could be that an equilibrium exists, whereby HEC induces the formation of a small cluster of silica particles. Based on the studies of these particles using different methods, we can propose a scheme for particle aggregation dependent on HEC content in the reaction mixture, illustrated in (Fig. 7).

## 4 Conclusions

Successful synthesis and functionalisation of silica nanoparticles decorated with HEC was achieved in a “one pot” reaction. The particles were fully characterised for size and surface functionality using a variety of physicochemical techniques. By comparing the size generated by DLS to that generated by TEM and SANS, a clustering effect of nanoparticles was observed, with increasing numbers of particles aggregating as a function of HEC concentration in the reaction. Given the low cost and high mass production of HEC in comparison to other polymers, this article provides evidence of a potential method to mass produce thiolated silica nanoparticles of a controlled size, suitable for a diverse range of applications.

## Conflicts of interest

The authors have no any conflicts of interests related to this publication.

## Supplementary Material

RA-008-C7RA08716K-s001
