# Supplementary material for: Structure and characterisation of hydroxyethylcellulose–silica nanoparticles
Source: RSC Adv. 2018 Feb 8;8(12):6471–8. doi: 10.1039/c7ra08716k (PMC9078266; doi:10.1039/c7ra08716k)
Supplement: RA-008-C7RA08716K-s001 [file RA-008-C7RA08716K-s001.pdf]

Electronic Supplementary Material (ESI) for RSC Advances.
This journal is © The Royal Society of Chemistry 2018

Structure and characterisation of hydroxyethylcellulose-decorated nanoparticles

Edward D.H. Mansfield,[a] Yash Pandya,[a] Ellina A. Mun,[a] Sarah E. Rogers,[b] Inbal Abutbul-Ionita,[c] Dganit Danino,[c] Adrian C. Williams,[a] and Vitaliy V. Khutoryanskiy[a]*

a School of Pharmacy, University of Reading, Whiteknights, Reading, Berkshire, UK, RG6 6AD, email: v.khutoryanskiy@reading.ac.uk

***b** ISIS Spallation Neutron Source, Science and Technology Facilities Council, Rutherford Appleton Laboratory, Harwell Science and Innovation Campus, Didcot, OX11 0QX U.K.*

***c** Technion – Israel Institute of Technology, Faculty of Biotechnology and Food Engineering*

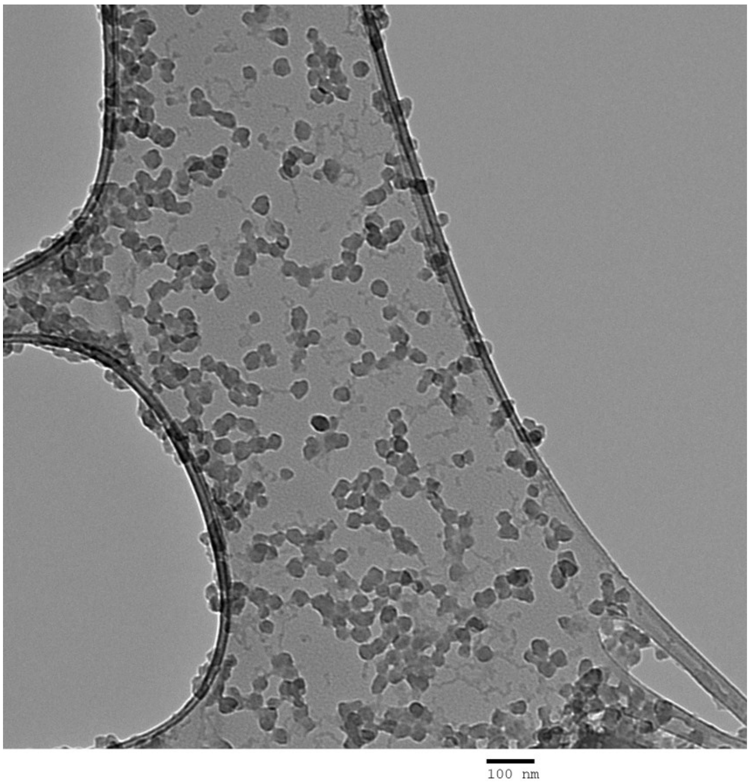

Y1

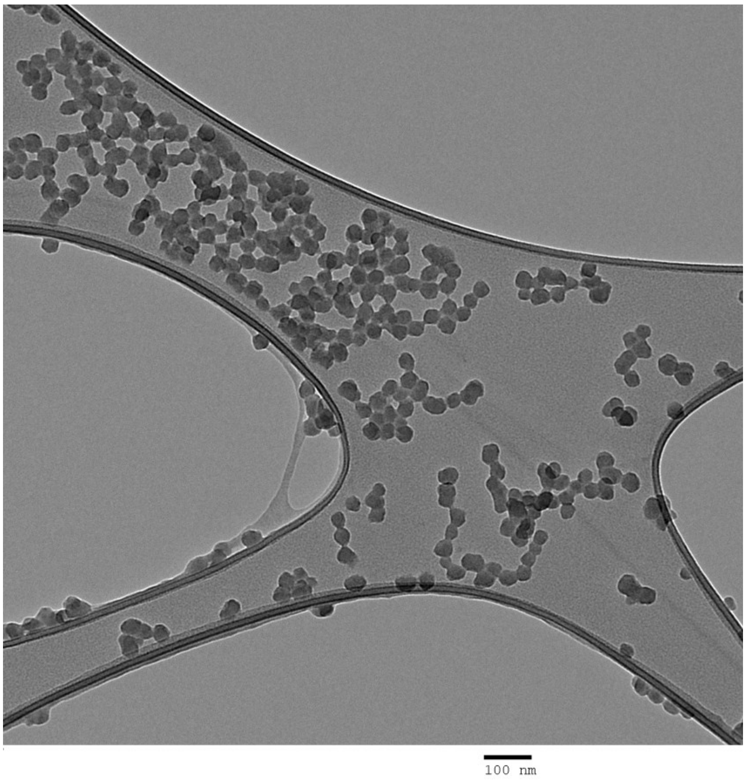

Y2

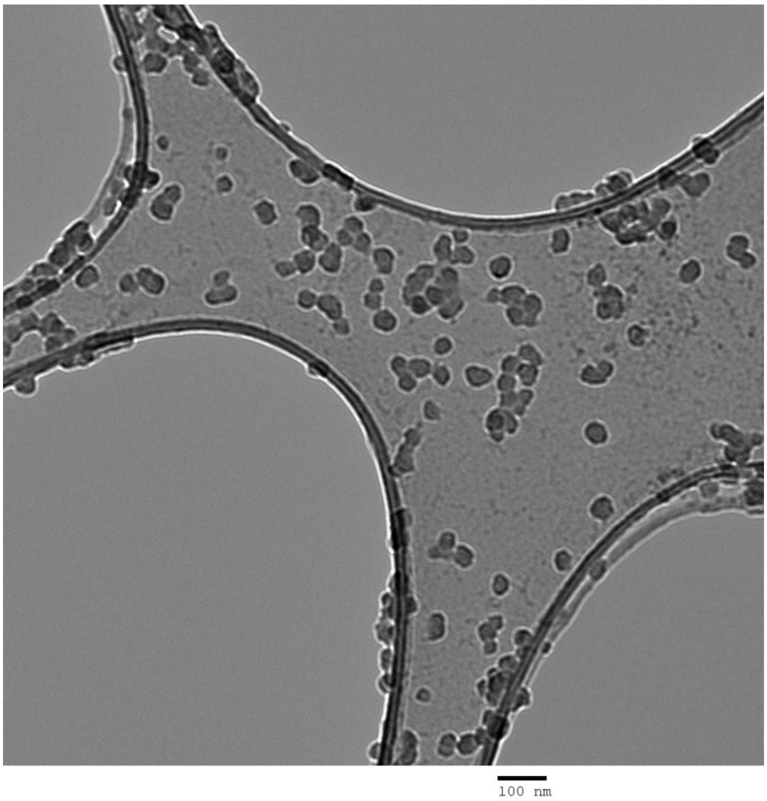

Y3

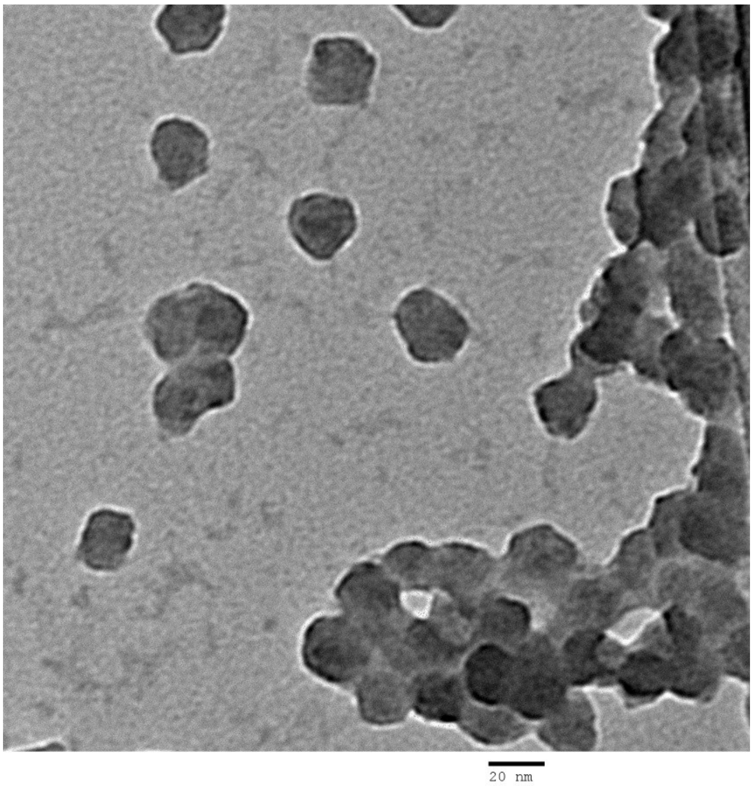

Y4

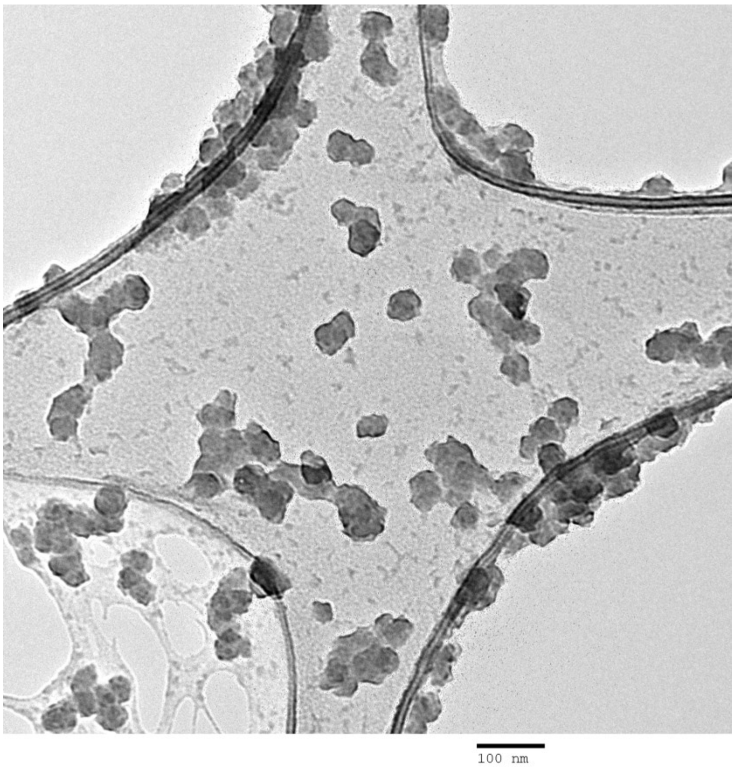

Y5

Figure SI1 Exemplar TEM images of nanoparticles

TGA analysis

To calculate the grafting density, the weight of polymer in the sample was divided by the surface area of the raw silica particle (based on the size determined by TEM/SANS analysis; 2827 nm^2). It should be noted that due to the presence of free HEC in the sample, there may be some error in this calculation.

Small Angle Neutron Scattering

Following collection and data reduction, all SANS data was modelled using the SASview software package (www.sasview.org).

Initially, data were modelled to a spherical form factor (Equation S1), however the results yielded a poor fit at higher q-values, suggesting a core-shell type structure. The likely reason for this is the presence of either free HEC or a shell consisting of HEC.

$$I(q) = \frac{scale}{V} \cdot \left[\frac{3V(\Delta\rho)(\sin(qr) - qr\cos(qr))^2}{(qr)^3}\right] + bkg$$

Equation S1

Where scale is volume fraction, V is the volume of the scattering object, r is the radius, bkg is the background, and Δρ is the scattering length density contrast factor (i.e. difference in SLD between the sphere and surrounding solvent).

To account for this addition, a core-shell sphere model was used (Equation S2).

$$P(q) = \frac{scale}{V_s}\left[3V_c(\rho_c - \rho_s)\frac{[\sin(qr_c) - qr_c\cos(qr_c)]}{(qr_c)^3} + 3V_s(\rho_s - \rho_{solv})\frac{[\sin(qr_s) - qr\cos(qr_s)]}{(qr_s)^3}\right]^2 + bkg$$

Equation S2

Where scale is a scaling factor, Vs is the volume of the outer shell, V_c is the volume of the core, r_s is the radius of the shell, r_c is the radius of the core, ρ_c is the SLD of the core, ρ_s is the SLD is the shell, ρ_{solv} is the SLD of the solvent, and bkg is the background. The fits for this model can be found in Fig SI2, and a table for the parameters in Table SI1. Given the uncertainty of how the HEC is interacting with the silica nanoparticles, the SLD parameter for the core and shell were left as floating variables, and SLD for solvent was fixed at $6.35x10^{-6}$ $Å^2$.

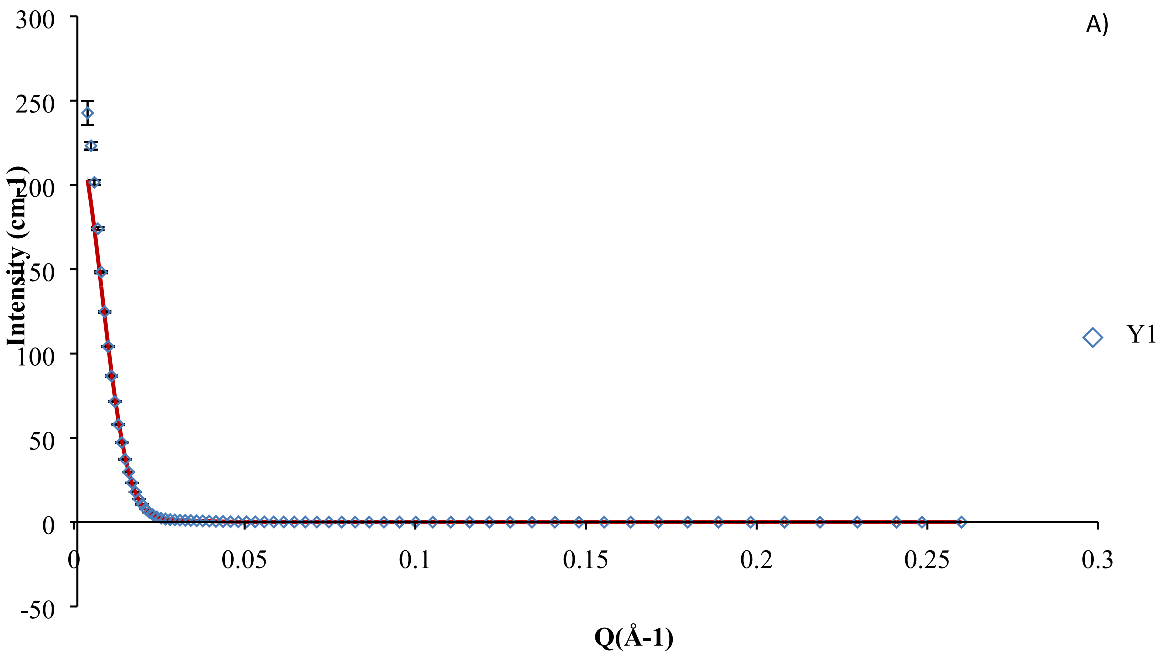

A)
Intensity (cm-1)
Q(Å-1)
Y1
300
250
200
150
100
50
0
-50
0
0.05
0.1
0.15
0.2
0.25
0.3

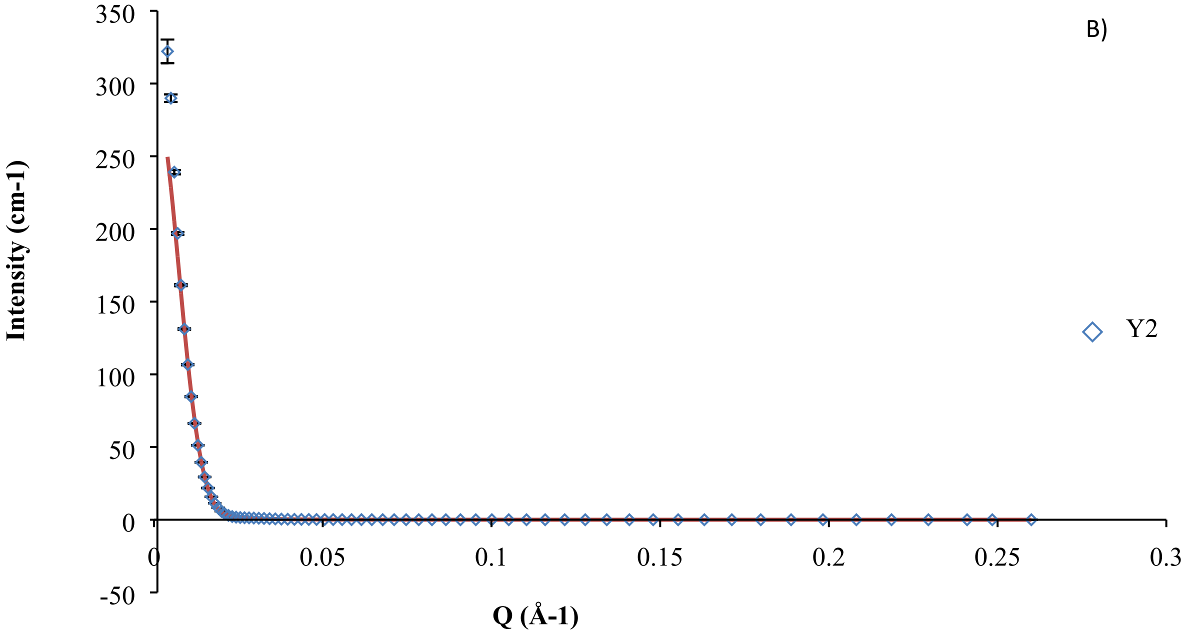

B)
Intensity (cm-1)
Q (Å-1)
Y2
350
300
250
200
150
100
50
0
-50
0
0.05
0.1
0.15
0.2
0.25
0.3

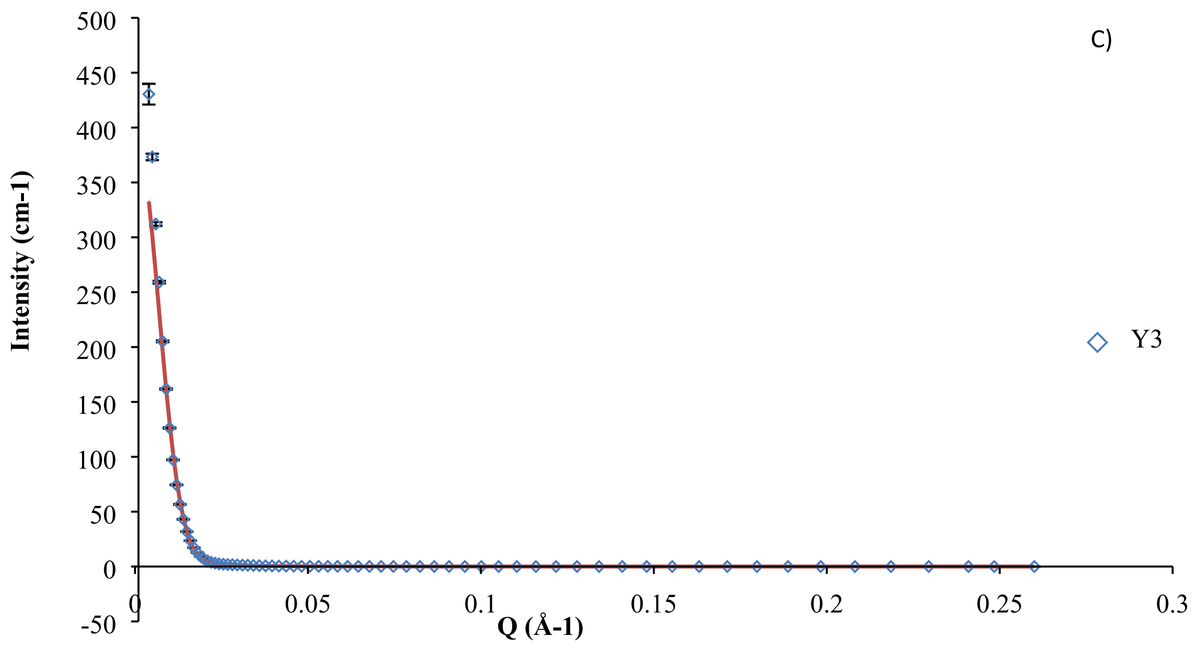
C)
Intensity (cm-1)
Q (Å-1)
Y3
500
450
400
350
300
250
200
150
100
50
0
-50
0
0.05
0.1
0.15
0.2
0.25
0.3

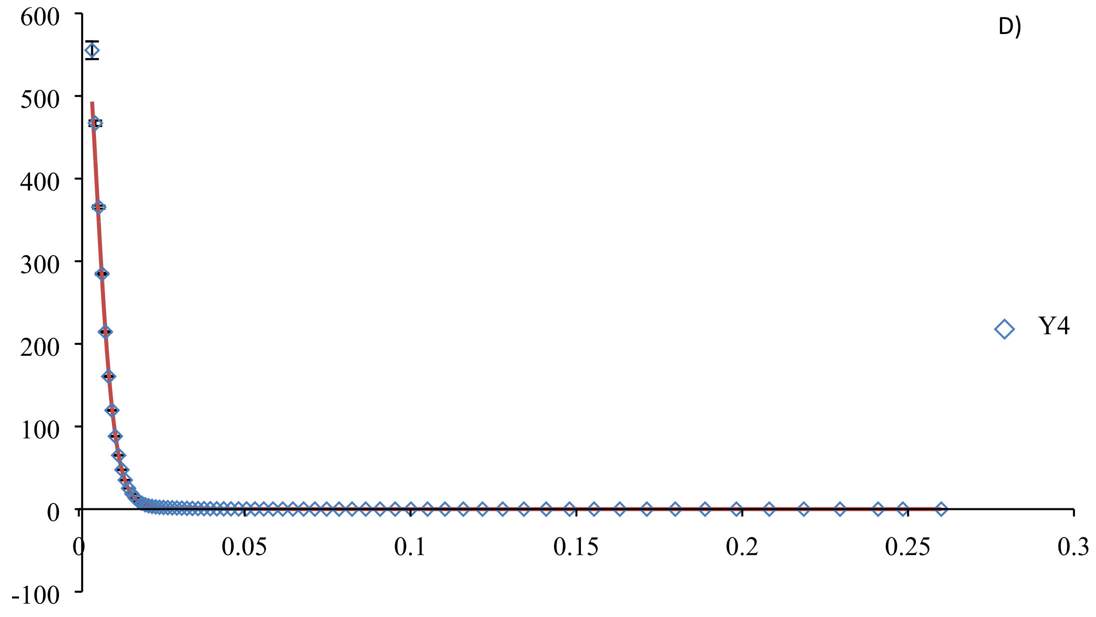
D)
Y4
600
500
400
300
200
100
0
-100
0
0.05
0.1
0.15
0.2
0.25
0.3

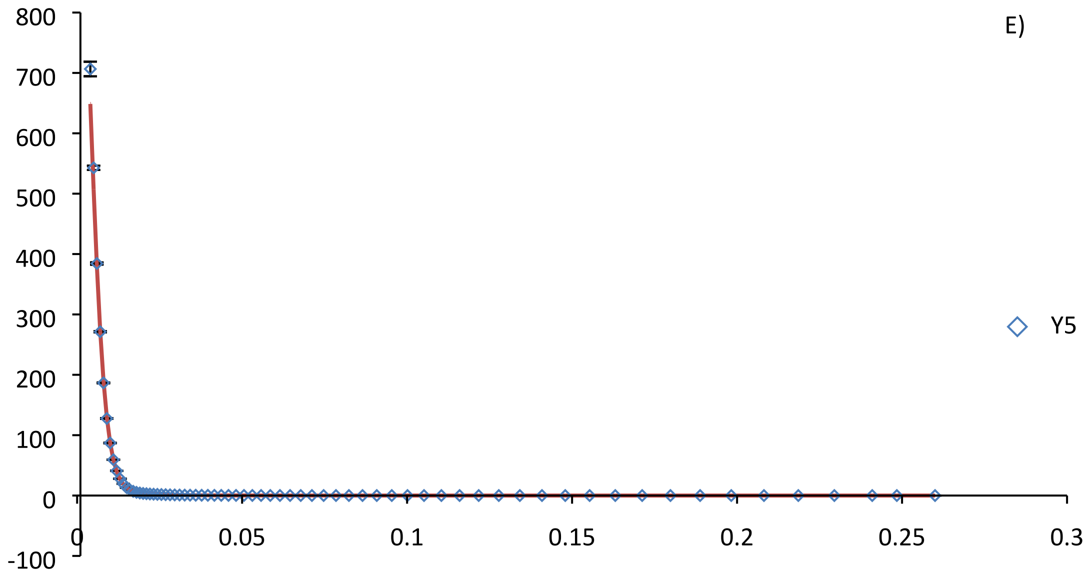

Fig SI2 Fitting to a core-shell model for HEC-silica nanoparticles. A) shows unfunctionalised silica, B) 0.1% HEC-silica, C) 0.5% HEC-silica, D) 1% HEC-silica, and E) 2% HEC-silica.

Table S1. Fitting parameters for Y1 to Y5 for the fits indicated in Fig SI3

	**Y1**	**Y2**	**Y3**	**Y4**	**Y5**
**Scale**	0.014	0.0.03	0.057	0.06	0.07
**Background (cm^{-1})**	0.065	0.069	0.065	0.068	0.07
**Radius (Å)**	152	172	179	146	175
**Thickness (Å)**	4.8	5.1	30	30	34
**SLD core (Å^2)**	$1.38x10^{-6}$	$4.6x10^{-6}$	$4.6x10^{-6}$	$3.7x10^{-6}$	$1.38x10^{-6}$
**SLD shell (Å^2)**	$1.38x10^{-6}$	$7.9x10^{-6}$	$6.7x10^{-6}$	$6.0x10^{-6}$	$5.2x10^{-6}$
**SLD solvent (Å^2)**	$6.35x10^{-6}$	$6.35x10^{-6}$	$6.35x10^{-6}$	$6.35x10^{-6}$	$6.35x10^{-6}$

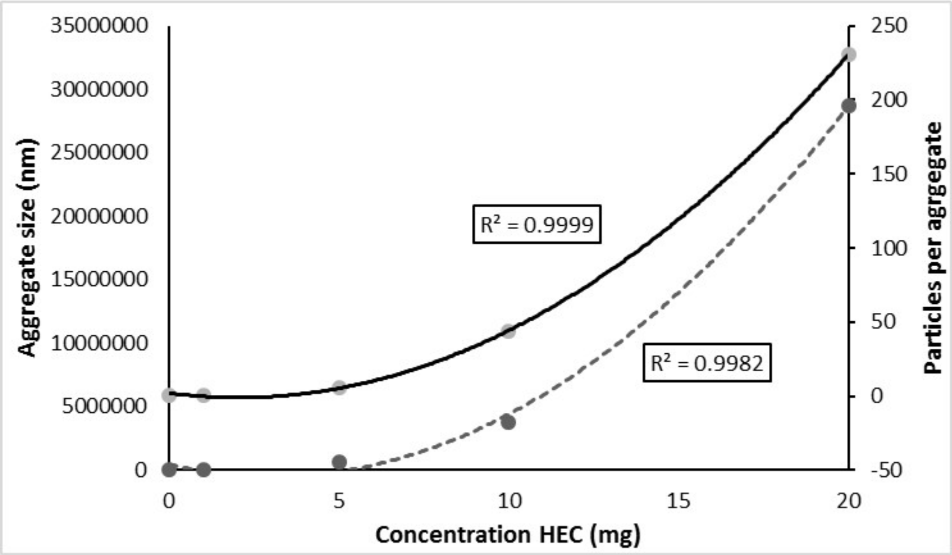

Fig SI3 Relationship between the concentration of HEC in the reaction mixture with aggregate size (dashed line) and number of hydrated particles per aggregate (solid line).

Estimation of number of particles per aggregate.

Using the aggregate size from DLS which includes the hydration shell around all of the particles in the aggregate, the volume of the hydrated aggregate can be calculated.

To estimate the number of particles in each aggregate, the hydrated aggregate volume from DLS is simply divided by the hydrated particle volume. Individual particle size data for each HEC concentration is available from the SANS or TEM diameters; SANS data was selected. However, SANS provides the sizes of the particle cores and so 20 nm (as the mean hydration layer thickness) was added to the SANS size data to account for the hydration shell we expect around each particle in the aggregate. From this, the volume of each hydrated particle was calculated. Dividing the hydrated aggregate volumes by hydrated particle volumes gave an estimate of the number of particles in the aggregate.

Clearly there are numerous assumptions in this approach. We assume the hydration shells of the particles within the aggregates remain at 20 nm thick and that the particles don't "share" the layer in the aggregates (i.e. the hydration shells may not be 20 nm between particles), we assume perfect dense packing and have taken SANS values rather than sizes from TEM which could provide some minor discrepancies. Not withstanding these caveats, the estimates illustrate that the higher HEC concentrations allows greater number of particles to aggregate.

Table SI2. Estimation of number of particles per aggregate

HEC concentration (%)	Aggregate Hydrodynamic radius by DLS (nm)	Aggregate Hydrodynamic volume by DLS (nm^3)	Particle diameter SANS (nm)	Estimated Hydrated Particle diameter SANS + 20 (nm)	Hydrated particle volume (nm^3)	Number of hydrated particles per aggregate
0	25	65450	31	51	69556	1
0.1	27	82448	35	55	87114	1
0.5	54	659584	41	61	118847	~6
1	97	3822996	35	55	87114	44
2	190	28730912	42	62	124799	230
